# Transcriptome sequencing and microarray development for the Manila clam, *Ruditapes philippinarum*: genomic tools for environmental monitoring

**DOI:** 10.1186/1471-2164-12-234

**Published:** 2011-05-12

**Authors:** Massimo Milan, Alessandro Coppe, Richard Reinhardt, Leonor M Cancela, Ricardo B Leite, Carlos Saavedra, Claudio Ciofi, Guido Chelazzi, Tomaso Patarnello, Stefania Bortoluzzi, Luca Bargelloni

**Affiliations:** 1 Department of Public Health, Comparative Pathology, and Veterinary Hygiene, Faculty of Veterinary Medicine, University of Padova, Viale dell'Università 16, 35020 Legnaro, Italy; 2 Department of Evolutionary Biology, University of Florence, 50125 Florence, Italy; 3 Biology Department, University of Padova, Via G. Colombo 3, I-35131 Padova, Italy; 4 Max Planck Institute for Molecular Genetics, Ihnestraße 63-73, 14195 Berlin, Germany; 5 CCMAR/University of Algarve, Campus de Gambelas, Faro, Portugal; 6 Instituto de Acuicultura de Torre la Sal (IATS), Consejo Superior de Investigaciones Cientificas (CSIC), 12595 Ribera de Cabanes, Castellon, Spain

## Abstract

**Background:**

The Manila clam, *Ruditapes philippinarum*, is one of the major aquaculture species in the world and a potential sentinel organism for monitoring the status of marine ecosystems. However, genomic resources for *R. philippinarum *are still extremely limited. Global analysis of gene expression profiles is increasingly used to evaluate the biological effects of various environmental stressors on aquatic animals under either artificial conditions or in the wild. Here, we report on the development of a transcriptomic platform for global gene expression profiling in the Manila clam.

**Results:**

A normalized cDNA library representing a mixture of adult tissues was sequenced using a ultra high-throughput sequencing technology (Roche 454). A database consisting of 32,606 unique transcripts was constructed, 9,747 (30%) of which could be annotated by similarity. An oligo-DNA microarray platform was designed and applied to profile gene expression of digestive gland and gills. Functional annotation of differentially expressed genes between different tissues was performed by enrichment analysis. Expression of Natural Antisense Transcripts (NAT) analysis was also performed and bi-directional transcription appears a common phenomenon in the *R. philippinarum *transcriptome. A preliminary study on clam samples collected in a highly polluted area of the Venice Lagoon demonstrated the applicability of genomic tools to environmental monitoring.

**Conclusions:**

The transcriptomic platform developed for the Manila clam confirmed the high level of reproducibility of current microarray technology. Next-generation sequencing provided a good representation of the clam transcriptome. Despite the known limitations in transcript annotation and sequence coverage for non model species, sufficient information was obtained to identify a large set of genes potentially involved in cellular response to environmental stress.

## Background

The Manila clam *Ruditapes philippinarum *(Adams & Reeve, 1850) is a bivalve mollusc of the family Veneridae native to the Indo-Pacific region. Because of its commercial value as seafood, this species has been introduced to other regions, where it has become permanently established. In Europe it was first imported in 1972 in France. Additional introductions occurred from Oregon to the United Kingdom, followed by numerous transfers within European waters for aquaculture purposes (Portugal, Ireland, Spain, and Italy). Natural reproduction of introduced individuals favored geographical expansion into the wild, particularly in Italy, France, Spain and Ireland where the Manila clam proved to be more resistant and grew faster than the endemic carpet-shell clam, *R. decussatus*. Consequently, *R. philippinarum *displaced its autochthonous congeneric species in most areas, and now represents the most important species for commercial clam landings in Europe. Globally, harvest of *R. philippinarum *has experienced a dramatic increase in the last 20 years, currently representing one of the major aquacultured species in the world (3.36 million metric tons in 2008). China is by far the leading producer (97.4% of total annual production) while Italy has a smaller but yet conspicuous production of over 65,000 tonnes per year [1].

Despite the relevance of Manila clam landings in world aquaculture, genomic resources for *R. philippinarum *are still extremely limited [2]. A small set of genetic markers is available [3] and only 5,707 transcripts has been sequenced and are already available on GENBANK. Although *R. philippinarum *is considered a robust species, capable of adapting to a wide range of environments, infectious diseases, chronic parasitic (*e.g. Perkinsus *-like microorganisms) and bacterial (*e.g*. brown ring bacterial disease) infections, it has been suffering mass mortality that have caused severe production losses in different areas (European Atlantic waters, Yellow Sea) [1]. The impact of infections is often aggravated under particular environmental conditions, such as extreme temperatures or limited availability of oxygen or nutrients. However, massive mortalities are rarely explained by a single parameter. An understanding of the interactions among different biotic and abiotic factors influencing survival is therefore a high priority for clam aquaculture. Functional genomics, or more specifically physiological genomics, *i.e. *a global analysis of transcriptome responses to different conditions, offers unprecedented opportunities to achieve such a goal. For instance, a genomic analysis was recently used to investigate summer mortality in the Pacific oyster [4]. To this end, the development of transcriptomic tools for the Manila clam is the first necessary step.

A second and possibly more important application of global gene expression profiling in *R. philippinarum *is environmental monitoring. Genomic technologies are increasingly used to evaluate the biological effects of various chemical pollutants on aquatic animals under either controlled conditions or in natural environments (*e.g. *[5,6]). While several hurdles remain to be overcome, the outlook for eco-toxicogenomics is extremely promising [7]. A sessile, filter-feeding organism living in the seafloor sediment, *R. philippinarum *represents an excellent "sentinel" species to assess the quality of marine environment. Two recent studies correlating different biochemical, cellular, and organismal markers with levels of pollutants in the sediment [8] or accumulated in the animals [9] support this view. However, a limited set of multiple biomarkers is usually employed in most of the studies. Therefore, a transcriptomic approach could provide a much broader analysis of different biological processes allowing for an integrated description of responses to xenobiotics [5,6].

The aim of the present study was to fill the gap in transcriptome sequence data available for the Manila clam and to develop a reliable and informative platform for global gene expression profiling, to be then applied to environmental monitoring. To this end, next-generation sequencing was coupled with a technology, in situ synthesized oligo array, which has provided a robust and flexible microarray platform in other species using conventional Sanger sequencing [10-18].

To date, 454 mollusc data are available only for *Mytilus galloprovincialis *and *Bathymodiolus azoricus *[19,20], and to our knowledge, this is the first report of an oligo DNA microarray developed using ultra-high throughput pyrosequencing in a mollusc species. A free web-accessible database including extensive transcript annotation and a blast search option was also developed in support of the gene expression platform.

In order to assess the feasibility of this newly developed *R.philippinarum *microarray to toxicogenomics, a preliminary investigation has been performed by profiling gene expression in gills and digestive glands of clams sampled in the industrial area of Marghera, a highly polluted site of the Venice Lagoon, compared to animals sampled in a clean area of the lagoon of Venice.

## Results and Discussion

### Next-generation sequencing and hybrid contig assembly

**Figure 1:**
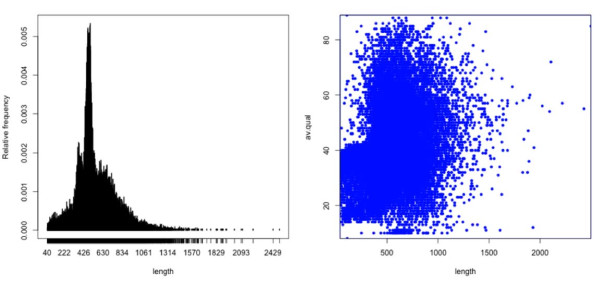
**Sequence length and average quality**. Distribution of sequence length and relationship between sequences length and average quality for the set of 32,606 contigs.

Starting from a total of 463,424 sequences (see Methods), a first run of hybrid assembly grouped 191,624 reads (41%) into 40,477 contigs. The resulting assembled sequences and the remaining singletons were then used as input for a second MIRA run (see methods) of assembly in order to produce meta-contigs from a fraction of partially redundant contigs obtained by the first run. This approach produced a set of 32,606 contigs. Summary statistics of the ESTs generated for *R. philippinarum *and their assembly are reported in Table 1. Figure 1 shows the distribution of sequence length and the relationship between length and average quality for the 32,606 contigs. All Roche 454 FLX reads have been deposited in GenBank (GenBank:SRR058508.1-SRR058508.457717).

**Table 1:** Summary of generated *Ruditapes philippinarum *ESTs and assembly results with statistics describing different properties of transcriptome contig sequences available in R*uditapes philippinarum *Database (compgen.bio.unipd.it/RuphiBase)

	Feature	Value
Sequences	454 reads	**457,717**
	Sanger sequences	**5,707**
	
	Assembled sequences	**191,624**

Contigs	First run of hybrid assembly	**40,477**
	
	Meta-contigs of second assembly	**4,990**
	Not re-assembled	**27,616**
	
	Total (*Ruditapes philippinarum *transcriptome)	**32,606**
	Mean length (bp)	**546**
	Max length (bp)	**2497**
	Mean Average quality (Phred)	**39.2**
	Mean GC%	**33.5**

### Transcriptome annotation

Putative identities of assembled contigs and meta-contigs were obtained by running Blastx and Blastn similarity searches on several protein and nucleotide databases (see Additional file 1). Of 32,606 unique sequences, 7,907 (24%) showed at least one significant match (e < 10-5) in the NCBI non-redundant protein database. The use of Blast2GO software allowed the association of one or more GO terms to 6,867 *R. philippinarum *data base entries. Of these, 2,788 were linked to "Biological Process" (BP) GO entries, 2,880 to "Cellular Component" (CC) entries, and 3,141 to "Molecular Function" (MF) entries. Unique GO terms represented in *R. philippinarum *entries were 1,515 for BP, 380 for CC, and 655 for MF. A simplified view of these GO terms using a "Generic GO Slim" showed 46 BP, 30 CC, and 34 MF classes (see Additional File 2).

In addition to the annotation with Blast2GO, Blast searches against UniProtKB/Swiss-Prot database, UniProtKB/TrEMBL database and 26 different species-specific data bases (see Additiona file 1) were implemented in order to further increase the number of putatively annotated *R. philippinarum *contigs (see Methods for details). This approach provided a significant match for additional 1,840 transcripts, which showed no previous correspondence with either the NCBI non-redundant protein or nucleotide database, and brought the final number of clam entries associated with a known protein or transcript to 9,747 (30%).

### RuphiBase, a Ruditapes philippinarum database

All 32,606 contig sequences as well as different layers of results for data analysis are available through RuphiBase [21], a free web-accessible database implemented using MySQL and Django web framework. RuphiBase is centered on contig sequence and annotation, and can be searched by contig ID and key word match on different textual fields. Moreover, it allows the user to conduct a local BLAST search on the fly against the transcripts database, in order to identify one or more transcripts significantly similar to a given query sequence. Indeed, massive and customizable data retrieval is provided by a browsing system. For each contig, a gene-like entry shows different data and bioinformatic analyses results according to the scheme detailed below:

• *Contig information*. For each contig, identified by an ID and a preliminary description, the FASTA sequence is given, along with an informative contig description, which is defined by the Blast2GO natural language text mining functionality, applied to BLAST hits description. The best hit is used when a BLAST2GO description is unavailable.

• *Assembly*. The list of reads belonging to the contig is given together with two FASTA files which include all read sequences, contig with reads and ESTs sequences and ACE format multiple alignment of the contig with reads and ESTs.

• *Gene Ontology*. GO terms associated to each transcript are given for BP, MF, and CC, with hyper-link to the GO database.

*BLAST results*. BLAST results, for both nucleotide and protein database searches, are shown in a dedicated section in the classic BLAST output format. These results are hyperlinked to external databases, and include the list of alignment descriptions and details about the pairwise alignments of each transcript with the corresponding BLAST hits.

### Microarray quality assessment

A total of seven microarray experiments (three biological replicates for gills and four for the digestive gland) were carried out. After data extraction, hybridization success for each probe was inferred if flag "glsFound" values was equal to 1 (see Methods). Across all experiments, only 131 probes (0.3%) never showed a signal higher than the background, while 19,360 probes (46%) were always successful and 37,379 (88%) were successful in at least four experiments.

**Figure 2:**
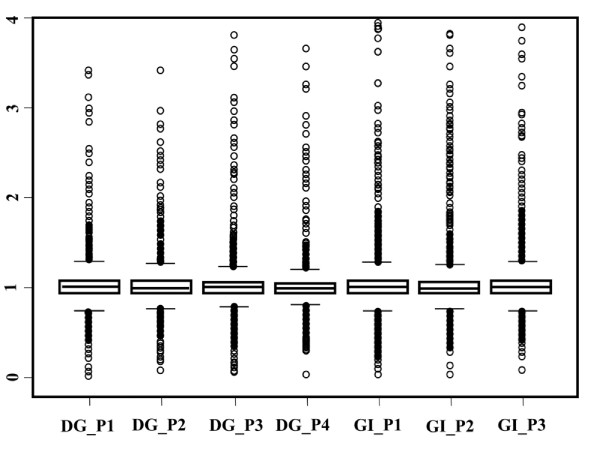
**Correlation between Probe_1 and Probe_2**. Distribution of observed fold-changes between Probe_1 and Probe_2 for each array experiment in the digestive gland (DG) and gill (GI) pools (P).

To evaluate the repeatability of the array results, microarray data for the digestive gland (four biological replicates, each replicate consisting of a pool of five individuals) were normalized. The degree of mutual agreement between replicates was assessed by estimating the Pearson correlation coefficients (r) on the entire set of expression values. Pairwise comparisons of replicate experiments showed correlation coefficients with r > 0.99 and were always significant (p-value < 0.01) (see Table 2), confirming a good reproducibility of the microarray platform. Normalized fluorescence data for these comparisons have been deposited in the GEO database [22] under accession numbers GEO:GSE24050. The Manila clam microarray platform is characterized also by the presence of two duplicated probes, at different coordinates on the same array, for a total of 2,000 annotated transcripts. The variability between two identical probes for the same transcript was evaluated using the ratio between the two probe intensity levels (fold-change, FC) as a measure of signal difference. This ratio is expected to assume a value around 1. In Figure 2 each box plot describes the distribution of observed fold-changes between Probe_1 and Probe_2 for each array experiment in the digestive gland and gills pools. This is symmetrical, centered around 1 and equal across all the experiments. Concordance of hybridization signal for probe pairs was confirmed by estimating Pearson correlation coefficients between Probe_1 and Probe_2 for each gene across seven experiments. The correlation coefficient was always greater than 0.95 and highly significant (p < 0.0001) (data not shown).

**Table 2:** Correlation coefficients on the entire set of expression values across biological replicates (**p-value < 0.01)

	Digestive gland Pool 1	Digestive gland Pool 2	Digestive gland Pool 3
**Digestive gland_Pool 2**	0.9718101**		
**Digestive gland _Pool 3**	0.9714377**	0.977098 **	
**Digestive gland _Pool 4**	0.9665312**	0.9789406**	0.981809**

### Comparison of gene expression in the digestive gland and gills

Fluorescence data microarray experiments of three biological replicates consisting of pooled digestive glands and three pools of gills sampled in Alberoni, a clean area in the Venice Lagoon, were normalized and used to identify genes that were differentially expressed in different tissues.

Digestive glands and gills were chosen because of their relevant role on detoxification of xenobiotics as well as filtration of suspended matter and as defense barrier, respectively. These are cellular/organismal processes crucial in the response to chemical pollutants and/or pathogens exposure. Processed data were deposited in the GEO database [22] under accession number GSE24101. A two-unpaired class Significance Analysis of Microarray (SAM) test was carried out on normalized data, enforcing a False Discovery Rate (FDR) of 3%. A list of 10,159 significant probes, corresponding to 8,512 unique transcripts, was obtained. A total of 2,880 transcripts were up-regulated in pooled samples of digestive gland compared to gills with a FC ranging from 3 to 23,550, while a total of 7,279 transcripts was up-regulated in the gills compared to the digestive gland with a FC ranging from 3 to 18,200. A putative annotation could be obtained for 3,491 genes that were differentially expressed in the two tissues (see Additional file 3). A more systematic functional interpretation of the set of differentially expressed genes was obtained by an enrichment analysis using the Database for Annotation, Visualization, and Integrated Discovery (DAVID) software [23] with two alternative strategies. In the first case, *R. philippinarum *entries were matched to human Ensembl Gene IDs, while in the second strategy *R. philippinarum *entries were associated with zebrafish Ensembl Gene IDs (see Methods). Human or zebrafish IDs corresponding to differentially expressed Manila clam transcripts and to all genes represented on the array were then used to define a "gene list" and a "background" in DAVID, respectively. This allows functional annotation of differentially expressed genes through enrichment analyses based on an integrated biological knowledgebase, containing over 40 annotation categories. The second strategy allowed the assignment of a putative homologue to a larger number of clam transcripts. In total, 406 genes up-regulated in the digestive gland and 660 genes up-regulated in the gills found a corresponding functional annotation in DAVID. Enrichment analysis for up-regulated transcripts in the digestive gland showed 5 KEGG (Kyoto Encyclopedia of Genes and Genomes) pathways, 20 Biological Process (BP) terms, 5 Cellular Component (CC) terms, and 16 Molecular Function (MF) terms to be significantly over-represented (see Table 3). Enriched gene sets were involved in typical liver and pancreas metabolic processes such as cytochrome P450-mediated metabolism of xenobiotics, retinol metabolism and glutathione metabolism. The digestive gland of molluscs is also called "hepatopancreas" and integrates functions that are liver- and pancreas-specific in vertebrates. A notch signaling pathway was found among enriched KEGG pathways, although with a low statistical significance value (*P *= 0.09). This pathway has a role in timely cell lineage specification of both endocrine and exocrine pancreas.

**Table 3:** GO terms significantly represented among up-regulated genes in digestive gland compared to gills tissue

	Term	Count	p-value	F.E.
**KP**	dre00982:Drug metabolism	8	3.9E-05	5.9
	dre00980:Metabolism of xenobiotics by cytochrome P450	8	3.9E-05	5.9
	dre00480:Glutathione metabolism	9	8.8E-04	3.7
	dre04142:Lysosome	14	1.9E-03	2.4

**BP**	GO:0055114~oxidation reduction	39	6.4E-06	2.0
	GO:0048468~cell development	15	6.7E-06	3.6
	GO:0040008~regulation of growth	11	7.2E-06	4.7
	GO:0001558~regulation of cell growth	10	3.2E-05	4.6
	GO:0009308~amine metabolic process	17	4.2E-05	2.9
	GO:0022008~neurogenesis	14	4.2E-05	3.3
	GO:0030182~neuron differentiation	12	4.6E-05	3.8
	GO:0048699~generation of neurons	13	8.2E-05	3.4
	GO:0016052~carbohydrate catabolic process	13	2.3E-04	3.1
	GO:0030154~cell differentiation	22	2.3E-04	2.2
	GO:0048869~cellular developmental process	22	8.4E-04	2.1
	GO:0005975~carbohydrate metabolic process	18	2.2E-03	2.1
	GO:0007399~nervous system development	14	2.5E-03	2.4
	GO:0006066~alcohol metabolic process	13	7.7E-03	2.2
	GO:0009653~anatomical structure morphogenesis	20	1.1E-02	1.8
	GO:0006508~proteolysis	29	1.2E-02	1.5
	GO:0006629~lipid metabolic process	12	1.3E-02	2.2

**CC**	GO:0005576~extracellular region	20	1.13E-05	2.88
	GO:0016021~integral to membrane	40	2.11E-05	1.85
	GO:0031224~intrinsic to membrane	40	2.44E-05	1.84
	GO:0016020~membrane	55	3.34E-05	1.57
	GO:0044425~membrane part	42	0.001042	1.54

**MF**	GO:0030246~carbohydrate binding	29	2.55E-15	4.56
	GO:0005529~sugar binding	21	2.06E-11	4.69
	GO:0003824~catalytic activity	142	4.26E-06	1.29
	GO:0016491~oxidoreductase activity	46	1.69E-05	1.81
	GO:0005509~calcium ion binding	27	5.43E-05	2.18
	GO:0008233~peptidase activity	30	5.02E-04	1.85
	GO:0070011~peptidase activity, acting on L-amino acid peptides	29	5.42E-04	1.87
	GO:0004175~endopeptidase activity	25	5.63E-04	1.99
	GO:0004197~cysteine-type endopeptidase activity	11	1.10E-03	3.04
	GO:0016787~hydrolase activity	59	2.99E-03	1.39
	GO:0008234~cysteine-type peptidase activity	11	3.79E-03	2.66
	GO:0004872~receptor activity	18	3.97E-03	2.01

Here are the Biological Process (BP), Cellular Component (CC) and Molecular Function (MF) represented by at least 10 genes. Significantly represented KEGG pathways (KP) are also reported. Gene count, p-value and fold enrichment (F.E.) corresponding to each term are indicated.

Enrichment analysis on genes that were up-regulated in gills showed 10 KEGG pathway terms, 36 BP-GO terms, 35 CC-GO terms and 11 MF-GO terms, all significantly over-represented (see table 4). Genes over-expressed in the gills were involved in different cellular functions, including cell proliferation, differentiation, and migration. Two signaling pathways, Wnt and JAK-STAT, appeared to be significantly over-represented. The Wnt signaling pathway, with 13 genes over-expressed in gills, describes a complex network of proteins with a broad role in embryogenesis as well as in several cell processes of adult animals. The JAK-STAT signaling pathway transduces information from various chemical signals outside the cell to transcriptional regulation and it is involved in a wide array of cell activities. Other significant pathways, RIG-I-like receptor, NOD-like receptor, and Toll-like receptor signaling suggest a relevant role of immune response for the gills. This is supported by the large amount of hemocytes present in the gills and the presence of lectins and lysozyme among differentially expressed genes (data not shown). Enrichment in genes involved in blood vessel and vascular development is expected in a highly vascularized tissue as the gills, while over-representation of microtubule-associated proteins might reflect the importance of cytoskeletal structures in gill epithelia.

**Table 4:** Biological Process (BP), Cellular Component (CC), KEGG pathways (KP) and Molecular Function (MF) significantly represented by at least 10 genes up-regulated in gills compared to digestive gland

	Term	Count	p-value	F.E.
**KP**	dre04510:Focal adhesion	20	1.05E-04	2.31
	dre04540:Gap junction	8	6.50E-03	2.93
	dre04310:Wnt signaling pathway	13	1.22E-02	2.02
	dre04210:Apoptosis	8	1.24E-02	2.69
	dre04114:Oocyte meiosis	9	1.48E-02	2.42
	dre04621:NOD-like receptor signaling pathway	7	3.09E-02	2.57
	dre04630:Jak-STAT signaling pathway	5	3.50E-02	3.36
	dre04622:RIG-I-like receptor signaling pathway	6	4.50E-02	2.69
	dre04620:Toll-like receptor signaling pathway	7	4.94E-02	2.35

**BP**	GO:0050794~regulation of cellular process	89	2.74E-04	1.35
	GO:0034622~cellular macromolecular complex assembly	16	3.69E-04	2.41
	GO:0065007~biological regulation	101	3.75E-04	1.31
	GO:0050789~regulation of biological process	91	8.68E-04	1.31
	GO:0007166~cell surface receptor linked signal transduction	22	1.16E-03	1.93
	GO:0034621~cellular macromolecular complex subunit organization	16	1.87E-03	2.15
	GO:0007017~microtubule-based process	11	2.48E-03	2.59
	GO:0022607~cellular component assembly	20	4.27E-03	1.84
	GO:0050896~response to stimulus	39	5.63E-03	1.47
	GO:0042981~regulation of apoptosis	10	6.07E-03	2.51
	GO:0065003~macromolecular complex assembly	16	6.68E-03	1.94
	GO:0032501~multicellular organismal process	60	7.63E-03	1.32
	GO:0009653~anatomical structure morphogenesis	28	9.94E-03	1.55
	GO:0042592~homeostatic process	17	1.01E-02	1.83
	GO:0001568~blood vessel development	10	1.07E-02	2.35
	GO:0043067~regulation of programmed cell death	10	1.07E-02	2.35
	GO:0010941~regulation of cell death	10	1.07E-02	2.35
	GO:0001944~vasculature development	10	1.07E-02	2.35
	GO:0044085~cellular component biogenesis	23	1.39E-02	1.60

**CC**	GO:0015630~microtubule cytoskeleton	11	0.005	2.423
	GO:0044430~cytoskeletal part	14	0.033	1.747
	GO:0005856~cytoskeleton	22	0.044	1.471

**MF**	GO:0003924~GTPase activity	11	5.20E-04	2.97
	GO:0005515~protein binding	85	3.75E-03	1.28
	GO:0003700~transcription factor activity	12	5.89E-03	2.26
	GO:0019001~guanyl nucleotide binding	22	6.17E-03	1.73
	GO:0005525~GTP binding	22	6.17E-03	1.73
	GO:0032561~guanyl ribonucleotide binding	22	6.17E-03	1.73

Gene count, p-value and fold enrichment (F.E.) corresponding to each term are indicated.

### Strand orientation and antisense transcripts

As already mentioned, a great majority of probes showed a higher-than-background signal in four or more experiments, and nearly all of them in at least one. Since for 16,052 transcripts two probes with opposite orientation were designed, bi-directional transcription appears a common phenomenon in the clam transcriptome. In fact, it is now clear that animal genomes are transcribed on both strands [24-26] and it is not to be excluded that part of the analyzed transcripts has a functional role [27]. In order to further explore this issue, microarray data for digestive glands and gills were analysed by examining sense-antisense probe pairs. After the exclusion of probes with missing data, absolute mean fluorescence signal values (f) obtained across biological replicates after normalization were divided into four classes (f < 10, 10 < = f < 100, 100 < = f < 1000, f < = 1000). Class assignment was conducted by considering the mean fluorescence value of the probe showing the lower signal for each pair comparison. Likewise, Fold Change (FC) between sense and antisense probes for each probe pair was assigned to four classes (FC < 1.5, 1.5 < = FC < 3, 3 < = FC < 10, FC > = 10). Our results showed that 75% and 73.5% of probe-pairs had a signal ratio >3 in the gills and digestive gland, respectively, and 60% reported a signal ratio >10 in both tissues (see Table 5). This suggested a prevalent strand-orientation for the majority of transcribed regions in the clam genome. On the other hand, the absolute fluorescence for the "minor" strand was greater than 100 for 1,267 (8.8%) and 985 (6.5%) probe-pairs in the gills and digestive gland, respectively. In addition, a signal ratio < 3 and a minimum of fluorescence >100 was recorded for 223 (1.6%) probe-pairs in the gills and 151 (1%) probe-pairs in the digestive gland. These results suggested that natural antisense transcripts (NATs) may be present in the clam transcriptome. Natural antisense transcripts have been originally identified by searching for EST collections, and appear to be widespread across species, although at different frequencies [28]. Various putative functions have been proposed for NATs [29]. For instance, an important role in the production of endogenous siRNAs is increasingly recognized [30]. A relevant question is whether NAT transcription is correlated, either positively or negatively, with the expression of their sense counterpart or it is independent of it. This was evaluated by analyzing gene/transcripts represented with both sense and antisense probes pairs with SAM, in order to identify those that were differentially expressed in gills and digestive glands (see Table 6). Setting a threshold for FC to 1.5 and enforcing a relatively stringent FDR (< 0.01), for 688 genes both probes presented a significant q value. Of these, 658 showed concordant direction in sense/antisense regulation, while for 30 genes the two probes were up-regulated in the gills and the digestive gland, respectively. Under a less stringent FDR (< 0.1), 3,042 probe-pairs resulted differentially regulated, with a proportion of paired probes expressed in opposite directions (0.05) similar to the one observed above (0.04)..

**Table 5:** Comparison between sense and antisense probes for each probe pair

	FC < 1.5	1.5 < FC < 3	3 < FC < 10	FC > 10	TOTAL
**GILLS**					

**total**	1,958	1,635	2,118	8,629	14,340
**F LS < 10**	1,683	1,197	1,319	4,920	9,119
**10 < F LS < 100**	194	296	592	2,872	3,954
**100 < F LS < 1000**	66	119	162	707	1,054
**F LS > 1000**	15	23	45	130	213

**DIGESTIVE GLAND**					

**total**	2,061	1,909	2,646	8,411	15,027
**F LS < 10**	1,783	1,451	1,936	5,530	10,700
**10 < F LS < 100**	227	358	583	2,174	3,342
**100 < F LS < 1000**	45	84	105	583	817
**F LS > 1000**	6	16	22	124	168

The number of probes belonging to each Fold Change (FC) classes (FC < 1.5, 1.5 < = FC < 3, 3 < = FC < 10, FC > = 10) is reported. Probes are also distinguished according to the mean fluorescence value of the probe showing the lower signal (FLS, Fluorescence Lower Signal).

**Table 6:** Gene/transcripts represented with both sense and antisense probe pairs differentially expressed between gills and digestive gland (FC threshold set to 1.5)

q-value	Discordant S-AS pairs	Concordant S-AS pairs	
		
		UP-regulated in digestive gland	UP-regulated in gills
**< 18%**	898	532	3,489
**< 10%**	176	195	2,671
**< 1.1%**	30	97	561

### Clam genomic markers for environmental monitoring

A wide array of biochemical, cellular, and whole-organism markers have been applied to evaluate the biological effects of different types of pollutants in aquatic animals and to assess the status of marine ecosystems [31,32]. For instance, over-expression of metallothioneins (MTs) has been associated with exposure to heavy metals, inhibition of acetylcholinesterase (AChE) with organophosphorous, pesticide exposure, and induction of Vitellogenin (Vg) proteins (egg-yolk precursors) with the presence of xenoestrogens (endocrine-disruptors).

In the *R. philippinarum *platform developed in this study at least four transcripts (ruditapes_c21946, ruditapes_c30181, ruditapes_c7664, ruditapes_c12315) that appear to be AChE precursors and ten different expressed sequences (ruditapes_lrc32058, ruditapes_lrc32676, ruditapes2_c61, ruditapes2_c830, ruditapes2_lrc2117, ruditapes2_lrc4331, ruditapes2_lrc4377, ruditapes2_lrc4388, ruditapes2_lrc5136, ruditapes2_lrc5747) coding for a putative metallothionein were incorporated into the microarray. Finally, a transcript (ruditapes_c16240) showing a significant match with invertebrate Vg proteins was also included. It is worth mentioning that the lack of a specific anti-Vg antibody for many species impairs direct measure of such biomarker, and only indirect estimates of Vg concentration can be obtained using an alkali-labile phosphate (ALP) assay.

At the cellular level, loss of lysosomal membrane integrity has been observed as a consequence of oxidative stress induced by several class of chemicals. Reduced lysosomal membrane stability is also linked to increased autophagy [33]. To which extent these biochemical and cellular markers might be mirrored by gene expression markers present in RuphiBase based on GO-CC annotation, 73 lysosomal proteins including several cathepsins and other hydrolases could be found in the current clam transcriptome. Of note is a putative homolog (ruditapes_c23093) for Autophagic Transcript 12 (ATG12), an ubiquitin-like modifier necessary for macroautophagy, while several RuphiBase entries match with p14/ROBLD3, which is part of a protein complex that recruits mTOR (Mammalian Target Of Rapamycin), a key negative regulator of autophagy, to the lysosome membrane [34]. Further studies may be conducted to test whether chemical pollutants affecting lysosomal stability can induce alterations in expression levels of lysosomal and/or autophagy-related proteins. Indeed, tributyltin chloride has recently been shown to inhibit mTOR in neuronal cells [35].

**Figure 3:**
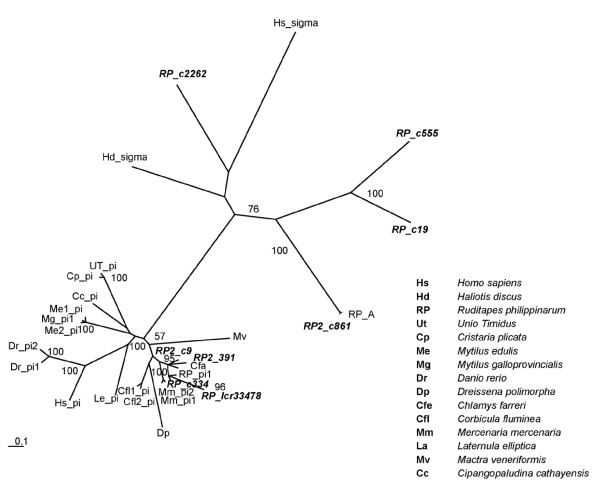
**Unrooted phylogenetic tree showing the relationships between published and unpuplished GST from *R. philippinarum *database (bold)**. Sequences are defined by two-letter species abbreviation followed by the GST symbol (pi or sigma) whenever possible. Bootstrap values are assigned to each interior branch. Values less than 50% are not shown. Genbank or Ensembl accession numbers are as follows: Hs_sigma (ENS:ENSP00000295256), HD_sigma (GenBank::AB026603.1), RP_A (GenBank:ACU832161), Mv (GenBank:ADB91399), Cfa (GenBank:ACL80138), MM_pi1 (GenBank:ABV29188), MM_pi_2 (GenBank:ABV29187), Dp (GenBank:ABP73387), Cfl1_pi (GenBank:ABO47816), Cfli2_pi (GenBank:AAX20374), Le_pi (GenBank:ABV44413), Hs_pi (ENS:ENSP00000381607), Dr_pi1 (ENS:ENSDARP00000004830), Dr_pi2 (ENS:ENDARP000000744422), Me1_pi (GenBank: AAF35893), Me_2pi (GenBank:AAS60226), Mg_pi1 (GenBank: AAM91994), Cc_pi (GenBank:ACJ03598), Cp_pi (GenBank:ADM88875), Ut_pi (GenBank:AAX20373), RP_pi (GenBank:ACM16805).

A separate discussion is required for GSTs, which constitute a large protein family [36], with a pivotal role in detoxification of xeno-compounds. These enzymes, involved in the conjugation of reduced glutathione to electrophilic centers on a wide variety of substrates, contribute to the detoxification of endogenous compounds (*e.g. *peroxidised lipids) as well as xenobiotics, and an increased GST activity has been observed after exposure to a broad set of pollutants. A total of 118 RuphiBase entries were annotated as putative GSTs of different subfamilies. Apart from four microsomal GSTs, putative cytosolic GSTs were divided into five subfamilies: 61 GST-σ, 21 GST-θ, 11 GST-π, 7 GST-μ, 4 GST-ω and 10 unclassified GST isoforms. Comparison of clam transcripts against single-species complete transcriptomes revealed the highest number of matches (69) with zebrafish., 33 α-like GSTs, 7 GST-μ, and 29 GST-π isoforms were identified. Human-clam comparison returned only 36 matches, although subfamily classification was more complex with 6 GST-μ, 10 GST-π, 1 GST-ξ, 15 GST-θ and 4 GST-ω isoforms. To further explore the incongruence between different annotations, we conducted a detailed analysis of the 29 GST-π isoforms classified on the basis of similarity against zebrafish. Eight transcripts showed an open reading frame (ORF) encoding a putative complete coding sequence for GST, while four transcripts presented an ORF encoding a partial GST coding sequence. The remaining sequences (17) contained either partial or complete GST coding sequences with reading frames interrupted by stop codons. Comparative sequence analysis revealed that these frame-shifts were always due to insertions/deletions (indels) within short homopolymeric stretches, a known problem with 454 pyrosequencing technology [37]. A phylogenetic tree was then reconstructed (see Figure 3) using the eight complete RuphiBase GSTs together with the best matching protein from GenBank as well as from human and zebrafish putative homologs (see Methods). It is clear that the classification based on comparison with zebrafish is incorrect, hiding two groups of sequences, one belonging to the σ subfamily, the other containing *bona fide *GST-π proteins. Using Blast results, the remaining 21 partial and/or interrupted ORFs were assigned to one GST sequence present in the tree in Figure 3, to obtain a classification of all 29 sequences originally assigned to the π subfamily. A tree-like representation is better suited to analyze and display the evolution of protein families or sub-families including a large number of multiple gene copies. The gene genealogy in Figure 3 is just an example of what is expected in case of a significant multiplicity is observed, as is the case of the the subset of GST-encoding transcripts analyzed here. To which extent different GST sequences reflect the presence of distinct GST loci in the clam genome? Pairwise comparison of best matching clam sequences across 29 GST-encoding transcripts (average length 742 bp, range 286-1130 bp) showed that one third of sequence differences (145 out of 14,042 surveyed bp, average 1%, range 0-3%) was due to indels, a likely consequence of sequencing errors, but the majority (269, average 1.9% range 0.1-11%) are nucleotide replacements, which are much less frequently observed with 454 pyrosequencing technology. For 15 sequences the closest match has 1% sequence divergence, for 11 more than 2%. Therefore, although part of the observed sequence diversity might be explained as different alleles of single GST loci, a substantial number of GST isoforms appear to be encoded by different genes. It should also be reminded here that this is a conservative estimate because in most comparisons only a fragment of the total sequence for each transcript, generally the one encompassing the coding region, was aligned (average 79%, range 33-100%). A similar problem of classification affects 21 sequences assigned to the θ GST subfamily, with 18 transcripts finding a *Pleuronectes platessa *(plaice) θ GST as their best match in SwissProt. This plaice θ GST has been recently re-assigned to a novel GST class, ρ [38]. Therefore, most clam sequences attributed to the θ class might actually belong to this specific GST protein group, similar to the only putative θ GST from a mollusk species isolated so far [39]. On the other hand, the remaining three sequences matched either a mammalian or a avian θ GST protein and might represent the first evidence of molluscan θ GSTs.

A correct classification of GST proteins is often difficult [36], but it is mostly important when correlating the expression of different GST-encoding genes with exposure to specific groups of environmental pollutants, as the various GST classes show diverse substrate specificities, catalytic properties, and tissue distribution.

### Gene expression profiling of Manila clam sampled in a polluted area of the Venice lagoon

The Venice lagoon, the largest in the Mediterranean sea, is characterized by the presence of complex mixtures of xenobiotics, derived from both industrial and domestic effluents, which reach higher concentrations in specific areas, mainly close to the industrial zone of Marghera. Gene expression profiles of digestive glands and gills from Manila clams harvested in a cleaner area (Alberoni) of the Venice lagoon were compared to the corresponding tissues of clams sampled within the industrial area. This area shows high levels of contamination with different xenobiotics, as confirmed in various studies [40] and it is currently restricted for clam harvesting.

For each tissue and comparison, raw and normalized fluorescence have been deposited in the GEO data base [22] under accession number GEO:GSE27194. A two-unpaired class SAM test was carried out separately for digestive glands and gills on normalized data, enforcing a False Discovery Rate (FDR) of 10% and Fold Change (FC) of 1.5.

Comparison of expression profiles between the two areas revealed a remarkably large number of differentially expressed transcripts in both tissues, respectively 1,127 in the digestive gland and 2,432 in the gills. A limited set of transcripts (99) showed differential expression in both tissues. Fold-change differences varied from -174- to 1,446-fold in the gills, with a prevalence for up-regulated transcripts (1,412) compared to down-regulated ones (1,020) in samples collected in the industrial area. This trend is reversed for transcripts displaying the strongest signal, as 93 probes showed FC > 5 (13 with FC > 10), whereas 120 ones presented FC < -5 (22 with FC > 10). In the digestive gland, FC ranged between -30- and 62-fold. A significant bias toward up-regulated transcripts (852, 75% of all differentially expressed sequences, binomial test p < 0.00001) was observed in animals sampled in the industrial area, a bias that was even stronger for transcripts showing FC larger than ± 5-fold (94 with FC > 5, 26 with FC < -5; binomial test p < 0.000001).

Putative annotations were obtained respectively for 321 digestive gland- and 830 gills-specific transcripts by comparison against the NCBI protein non redundant database. When using the zebrafish transcriptome as a reference, respectively 247 (digestive gland) and 730 (gills) differentially expressed sequences could be associated with one *D. rerio *Ensembl Gene IDs (see Addition file 4).

In a comparison between natural population samples different environmental and/or physiological factors can influence gene expression profiles. The objective of the present study was to assess the role of chronic exposure to high levels of chemical pollution. To control for the effects of other factors, histological examination of collected animals was carried out showing similar sex ratio (1:1), comparable levels of parasitic contamination, average size (12.3 gr vs 14 gr), and reproductive stage (data not shown). Water temperature and salinity showed no significant differences between the two analyzed areas. Indeed, the temperature and salinity recorded at the time of sampling were 18°C and 32 ‰ and 20°C and 34‰ in Marghera and Alberoni respectively. Likewise, it seems quite difficult that strong genetic differentiation occurs at a such a small geographic scale (few kilometres), in the presence of a planktonic larval phase and a sustained water circulation within the Venice lagoon. Although evidence on population genetics for the Manila clam is limited, it has been shown that no genetic structure was present across four population samples in the Adriatic Sea, including the Venice lagoon [41].

Systematic functional annotation of differentially expressed transcripts, carried out through enrichment analysis in DAVID (see Methods) confirmed a putative role of pollution in the regulation of gene expression in the examined samples, especially in the digestive gland. This organ has been generally associated with response to pollutants, particularly with detoxification of xenobiotics. Three significantly enriched GO_BP terms (see Table 7), response to organic substances (GO:0010033), to cadmium ion (GO:0046686), and to methylmercury (GO:0051597), two enriched KEGG pathways, drug metabolism (dre00982) and metabolism of xenobiotics by cytochrome P450 (dre00980), support the evidence that the digestive gland is responsible for detoxification of environmental pollutants and suggests it as a target organ for the detection/identification of biomarkers of pollution. Manual annotation of significant transcripts identified additional genes in the digestive gland with a known role in the response to environmental pollution.

**Table 7:** GO terms significantly over-represented, among genes differentially expressed, between Alberoni and Marghera samples, in both gills and digestive gland

DAVID analysis of digestive gland differential expressed genes				
**Category**	**Term**	**Count**	**p-value**	**F.E.**

BP	GO:0007018~microtubule-based movement	4	0.014259	6.969072
	GO:0007017~microtubule-based process	5	0.021639	4.35567
	GO:0010033~response to organic substance	4	0.040861	4.878351
	GO:0046686~response to cadmium ion	3	0.078833	6.097938
	GO:0051597~response to methylmercury	3	0.078833	6.097938

MF	GO:0005856~cytoskeleton	11	0.008012	2.50387
	GO:0015630~microtubule cytoskeleton	5	0.028246	4.021368
	GO:0005874~microtubule	4	0.030885	5.361823
	GO:0045259~proton-transporting ATP synthase complex	4	0.09956	3.446886

KP	dre04510:Focal adhesion	8	0.035313	2.421429
	dre00982:Drug metabolism	4	0.042809	4.708333
	dre00980:Metabolism of xenobiotics by cytochrome P450	4	0.042809	4.708333

**DAVID analysis of gills differential expressed genes**				

**Category**	**Term**	**Count**	**p-value**	**F.E.**

BP	GO:0044267~cellular protein metabolic process	75	0.002721	1.314444
	GO:0006412~translation	43	0.004311	1.463856
	GO:0045333~cellular respiration	10	0.008891	2.490526
	GO:0015980~energy derivation by oxidation of organic comp.	10	0.008891	2.490526
	GO:0034645~cellular macromolecule biosynthetic process	54	0.01233	1.323979
	GO:0019538~protein metabolic process	90	0.012991	1.209886
	GO:0006091~generation of precursor metabolites and energy	18	0.037073	1.607094
	GO:0006457~protein folding	13	0.046318	1.7576
	GO:0010467~gene expression	56	0.063065	1.210009
	GO:0044237~cellular metabolic process	125	0.063116	1.103545
	GO:0009060~aerobic respiration	5	0.065749	2.9575

MF	GO:0003735~structural constituent of ribosome	38	4.49E-04	1.689704
	GO:0005198~structural molecule activity	43	7.11E-04	1.593361
	GO:0015078~hydrogen ion transmembrane transporter activity	14	0.035716	1.766618
	GO:0016859~cis-trans isomerase activity	7	0.057275	2.334459
	GO:0003755~peptidyl-prolyl cis-trans isomerase activity	7	0.057275	2.334459
	GO:0008092~cytoskeletal protein binding	12	0.07344	1.697789
	GO:0015075~ion transmembrane transporter activity	23	0.07676	1.394612
	GO:0003924~GTPase activity	6	0.09088	2.334459

KP	dre03010:Ribosome	39	9.90E-07	2.003182
	dre04260:Cardiac muscle contraction	13	0.007105	2.136727
	dre00630:Glyoxylate and dicarboxylate metabolism	4	0.094374	3.287273

Details about "Biological process", "Molecular function" and "KEGG pathways" represented by at least 2 genes up regulated in each tissue.

Four transcripts encoding MTs (ruditapes2_lrc4377, ruditapes_lrc32058, ruditapes2_c830, ruditapes2_lrc4331) and two encoding sulfotransferase (SULT) (ruditapes_c20565, ruditapes_c28883) (see Additional file 4) are over-expressed in samples from the industrial zone. MTs provide protection against metal toxicity, are involved in the regulation of physiological metals (Zn and Cu) and provide protection against oxidative stress. MTs can be induced either by essential metals (Cu and Zn) or non-essential ones (Cd, Ag and Hg) in both vertebrates and invertebrates. Increased levels of MTs after experimental exposure to high Cu concentrations had been already reported in the digestive gland of *R. philippinarum *[42], while higher MT protein expression had been found in clams collected at sites nearby the industrial zone of Marghera [43-45].

SULTs, a family of phase II detoxification enzymes, are involved in the homeostasis of endogenous compounds as well as in the protection against xenobiotics. It is well known that sulfated products of environmental xenobiotics are more water-soluble and easily excreted from the body. Channel catfish (*Ictalurus punctatus*) exposed to Polycyclic aromatic hydrocarbons (PAHs) showed a marked induction of phenol-type sulfotransferase enzyme activity [46]. In addition, SULT1 was up-regulated in *Gadus morhua *male sampled in two contaminated sites of western Norway [47]. Although these genes play a documented role in the defense from chemicals [48], to our knowledge they have never been proposed as biomarkers in bivalve species.

AChE enzymatic activity is inhibited in response to organophosphate insecticides and exposure to other pollutants. Eight different clam transcripts encoding a peptide with putative cholinesterase activity are represented in the *R. philippinarum *microarray.

In the present study, an AChE-encoding gene (ruditapes_c12315) was over-expressed in both gills and digestive glands of clams sampled in Marghera. A similar finding has been already reported by *Somnuek *et al. (2009) [49], who demonstrated up-regulation of AChE gene expression in hybrid catfish exposed to chlorpyrifos and proposed this gene as biomarker for detecting the effects of organophosphate insecticides. The apparently opposite transcriptional response on AChE gene expression likely represents a compensatory modification to counteract inhibition of enzyme activity after xenobiotic exposure.

Several GST-coding transcripts were also found up-regulated in samples collected in the polluted area. Glutathione S-transferase (GST) catalyses the conjugation of reduced glutathione to electrophilic centers on a wide variety of substrates. This activity detoxifies endogenous compounds (*e.g. *peroxidised lipids) as well as xenobiotics and an increased of GSTs activity has been observed after exposure to a broad set of xenobiotics.

GST-coding genes that are over-expressed in clams sampled in Marghera either in the gills or in the digestive gland are different, except for a single transcript, which is up-regulated in both tissues (Table 8). Tissue-specific expression and sensitivity to dose/type of chemicals has been already reported in bivalves [50,51], suggesting a complex regulation of these effectors in the response to toxicants. Results obtained in the present study show also that various GST classes/isoforms are putatively involved in response to toxicants and emphasize the need for a proper classification of GST-coding genes. Five classes of cytosolic GSTs are differentially regulated together with a microsomal isoform in samples from the industrial area. Of special interest are two distinct genes, both encoding a GST-θ isoform. As mentioned previously GSTs belonging to the θ class have never been isolated in molluscs, and GSTθs apparently represent a numerically minor component of the GST arsenal in the Manila clam (3 putative θ isoforms among over 100 GST-encoding transcripts), yet two GSTθ-like genes showed marked up-regulation in putatively contaminated samples of the same species (see Table 8). GST-like activity is one of the most relevant biochemical parameters that are measured in environmental studies on chemical pollution, commonly using 1-choloro-2,4-dinitrobenzene (CDNB) as a substrate [52,53]. GST-θ enzymes, however, have a peculiar substrate specificity. GST- θ1 KO mice showed no difference for *in vivo *processing of CDNB, while GST-activity against other substrates [1,2-epoxy-3-(p-nitrophenoxy) propane (EPNP), dichloromethane (DCM), and 1,3-bis(2-chloroethyl)-1-nitrosourea (BCNU)] was significantly lower after GST- θ 1 gene deletion. The results reported in the present study suggest that measuring GST-like enzymatic activity might not completely represent that complex GST-based response to toxicants in bivalves. Accurate characterization of GST-encoding genes in species that are used for environmental monitoring coupled with transcriptome analysis could provide a more precise analysis of such a response, differentiating tissue-specificity and disentangling GST isoform diversity.

**Table 8:** Up-regulated GST coding transcripts found up-regulated in samples collected in the polluted area of Marghera

Contig	GST subfamily	Tissue	Fold Change
ruditapes_s39905	σ	gills	4.2
ruditapes_lrc39890	ρ	gills	1.9
ruditapes_lrc31893	μ	gills	1.8
ruditapes_c17817	θ	gills	5.1
ruditapes_c37712	π	gills	2.2
ruditapes_s39905	σ	digestive gland	4.2
ruditapes2_c352	μ	digestive gland	1.6
ruditapes2_c567	θ	digestive gland	2
ruditapes2_c72	microsomal	digestive gland	3.3

## Conclusions

Whole-transcriptome analysis holds the promise to shed light on the genetic mechanisms underlying cellular and organismal response to physiological and pathological conditions (environmental stress, infections, chemical pollution). This is of particular importance for improved shellfish aquaculture and for cost-effective environmental monitoring. The aim of the present paper was to lay the foundations for transcriptomics in the Manila clam. To which extent this goal has been achieved? As demonstrated in previous studies [54], the use of next-generation sequencing technology yielded a number of expressed sequences unattainable until only recently. In our study, sequence assembly, annotation and development of a dedicated database resulted in a searchable, functionally annotated transcriptome for *R. philippinarum *(RuphiBase), which was then used to design a species-specific *in-situ *synthesized oligo microarray. This genomic platform has proven to provide reliable and highly reproducible results for global gene expression profiling [10-18]. Moreover, validation of the clam oligo microarray showed tissue-specific expression profiles and highly significant correlations across biological replicates. The current version of RuphiBase appears to offer already a good representation of the clam transcriptome, as shown by the broad array of potential markers of response to xenobiotics. Of particular relevance is the large number (>100) of GST-encoding transcripts observed in the Manila clam, which suggested a potential relationship between filter-feeding behaviour, ability to cope with high levels of pollution and availability of a wide array of detoxifying enzymes. The possible use of this microarray platform for toxicogenomic studies has been also demonstrated by comparative analysis of digestive glands and gills pool of Manila clam sampled in areas with different levels of chemical pollution of the Venice Lagoon.

On the other hand, despite the use of ultra-high throughput sequencing on normalized cDNA libraries constructed from all adult tissues, representation of the clam transcriptome is still incomplete. For instance, the signaling pathway for autophagy consists of at least 18 different components [55], yet only one of these, ATG12, a protein involved in autophagic vescicle assembly, was identified. The problem of incomplete representation of protein-coding transcripts will likely be solved in the near future, when reduction of sequencing costs and an increase in sequencing throughput will allow a much deeper sequence coverage even for non-model species transcriptomes. A more difficult issue to solve is the limited percentage of clam transcripts that can be matched against a known protein-coding gene. The large phylogenetic distance of the phylum Mollusca from other metazoan model species (*e.g. Drosophila melanogaster, Caenorhabditis elegans, Danio rerio, Mus musculus, Homo sapiens*) greatly reduces the power of a comparative approach for functional annotation. The only molluscan genome sequenced so far is that of *L. gigantea*, a gasteropod snail, which is functionally and evolutionarily distant from the class Bivalvia.

To conclude on a positive note, the next "call on (genomic) stage" is for the Pacific oyster, *Crassostrea gigas*. For this bivalve mollusk species, a high quality draft genome sequence is expected in 2011 thanks to the efforts of the Oyster Genome Consortium. Furthermore, worldwide aquaculture production of oysters amounts to over 4 million metric tons. The economic importance of the Pacific oyster has fuelled a large number of studies on the ecology, physiology, immunology, and genetics of *C. gigas *populations, and the possibility of targeted gene knock down has been recently demonstrated [56]. The opportunity of having a bivalve model species available would allow a more accurate genome annotation for other important molluscs such as the Manila clam.

## Methods

### Sampling, cDNA library costruction and sequencing

Samples of *R. philippinarum *were bought in a local market in Faro. In order to improve RNA representatively, clams were stressed by submitting them to quick changes of temperature and salinity prior to be sacrificed. Total RNA was extracted from all tissues of 20 individuals using the acid guanidinium thiocyanate-phenol-chloroform method [57].

Two libraries were constructed, one using a mixture of adult tissues and a second one using gonadal tissues and 2 to 4 mm long larvae.

A cDNA library was constructed using equal amounts of RNA and normalized for sequencing. The SMART (Switching Mechanism At 5' end of RNA Template) kit from BD Biosciences Clontech was used to construct the cDNA libraries which were later normalised using the duplex-specific nuclease (DSN) method [58].

Approximately 15 μg of normalized cDNA were used for sequencing library construction at the Max Planck Institute, following procedures described in [59]. Sequencing was performed using GS FLX Titanium series reagents and using one single region on a Genome Sequencer FLX instrument. Bases were called with 454 software by processing the pyroluminescence intensity for each bead-containing well in each nucleotide incorporation. Reads were trimmed to remove adapter sequences.

### Contigs assembly

A total of 457,717 sequence reads were produced using Roche 454 FLX technology from the normalized cDNA library constructed using a mixture of adult tissues (see above). The same library was previously used to obtain 2,866 ESTs with Sanger sequencing. An additional set of 2,790 ESTs was available from a second normalized cDNA library (whole larvae and adult gonads). In addition, 51 mRNA sequences available in NCBI (as to 11th November 2009) for *R. philippinarum *were available.

454 Sequence reads and all previously ESTs accessible in the NCBI database were then assembled into contigs, representing putative transcripts, by using a custom procedure based on two runs of MIRA3 assembly [60] and quality-based filtering. All contigs obtained with the first run of hybrid assembly were used for a second run to eliminate contig redundancy. Threshold values on contigs length and sequence quality were then applied to obtain a final set of contigs representing *R. philippinarum *transcripts.

### Transcripts annotation

The Basic Local Alignment Search Tool (BLAST) was used to perform annotation of *R. philippinarum *contigs. Batch Blast similarity searches for the entire set of contigs were locally conducted against NCBI (National Centre for Biotechnology Information) amino acidic non redundant (nr) database (release of October 4 2009) using Blastx option. Alignments with an E-value of at most 1 E-3 were considered significant and up to 20 hits per contig were taken into account.

To improve the number of annotated contigs five different approaches were attempted (see Additional file 1): i) blastx searches (cut off e-value of < 1.0 E-3) against protein database UniProtKB/SwissProt and UniProtKB/TrEMBL [61], ii) blastx (cut off e-value of < 1.0 E-3) and blastn (cut off e-value of < 1.0 E-5) searches against proteins and high quality draft trascriptomes of *Danio rerio, Gasterosteus aculeatus, Oryzias latipes, Takifugu rubripes, Tetraodon nigroviridis, Homo sapiens, Drosophila melanogaster *available on Ensembl Genome Browser (release 56) [62], iii) blastx (cut off e-value of < 1.0 E-3) and blastn (cut off e-value of < 1.0 E-5) searches against proteins, transcripts and assembly scaffolds of *Lottia gigantea *v1.0 database [63], iv) blastn search (cut off e-value of < 1.0 E-5) against *D. rerio, L. gigantea, O. latipes, T. rubripes, Salmo salar, H. sapiens, Oncorhynchus mykiss *databases stored in NCBI UniGene database [64], v) blastn search (cut off e-value of < 1.0 E-5) against *Crassostrea gigas *transcripts database [65] and *Argopecten irradians *EST database [66].

The Gene Ontology (GO) terms associations for "Biological process", "Molecular function" and "Cellular component" were performed using Blastx algorithm against the NCBI amino acid nr database implemented in Blast2GO software [67]. The "Generic GO slim" [68] set of the CateGOrizer program [69] was used to have an overview of the gene ontology content by simplifying the results of the GO annotation.

### DNA microarray design

Probe design started with selection of target sequences to be represented onto the *R. philippinarum *microarray. All annotated entries (9,747) were included. Non annotated transcripts were considered only if sequence length was ≥400 bp and average Phred sequence quality was ≥30, yielding 24,291 target sequences. As most sequence reads were obtained from a non directional cDNA library, sense strand orientation was inferred putatively from that of homologuous protein sequences of other species (see Methods).

One probe for annotated transcripts with known orientation was designed to construct a high-density oligo-DNA microarray, while two probes with both orientations were designed for contigs with ambiguous orientation. The same strategy was applied to unknown unique transcripts. For 8,239 contigs, the putative orientation was unambiguous across different databases and a single sense probe was designed. Two probes with opposite orientation (sense and antisense) were designed for a fraction of clam annotated transcripts (1,508 contigs) with ambiguous putative orientation and for non annotated sequences (14,544). Probe design was carried out using the Agilent eArray interface [70], which applies proprietary prediction algorithms to design 60 mer oligo-probes. Microarrays were synthesized *in situ *using the Agilent ink-jet technology with a 4 × 44 K format. Each array included default positive and negative controls.

A total of 40,332 out of 40,343 (99.9%) probes, representing 24,281 *R. philippinarum *transcripts were successfully obtained. Of these, 2,000 probes designed on known-orientation transcripts, were synthesized in duplicate on the array in order to test for *"reproducibility-within-array"*. The percentage of annotated transcripts represented on the microarray was 40.1%. Probe sequences and other details on the microarray platform can be found in the GEO database [22] under accession number GEO:GPL10900.

### Sample collection, RNA extraction, labeling and hybridization

The common bivalves *R. philippinarum *were collected during autumn 2009 in two different areas of Venice Lagoon characterized by different levels of environmental pollutants: Marghera and Alberoni (see Additional file 5).

Digestive gland and gills were dissected from 20 Manila clamsfor each sampling area. Four and three independent pools, for digestive gland and gills respectively, each consisting of 5 digestive gland or gills, were created.

Total RNA was extracted from pooled tissue samples using the RNAeasy Mini Kit (Qiagen, Hilden, Germany) following the manufacturer's instructions. RNA concentration was determined using a UV-Vis spectrophotometer, NanoDrop^® ^ND-1000 (NanoDrop Technologies, Wilmington, USA). RNA integrity and quality was finally estimated on an Agilent 2100 Bioanalyzer (Agilent Technologies, Palo Alto, CA).

Sample labeling and hybridization were performed according to the Agilent One-Color Microarray-Based Gene Expression Analysis protocol. Briefly, for each pool 200 ng of total RNA were linearly amplified and labeled with Cy3-dCTP. A mixture of 10 different viral poly-adenilated RNAs (Agilent Spike-In Mix) was added to each RNA sample before amplification and labeling, to monitor microarray analysis work-flow. Labeled cRNA was purified with Qiagen RNAeasy Mini Kit, and sample concentration and specific activity (pmol Cy3/μg cRNA) were measured in a NanoDrop^® ^ND-1000 spectrophotometer. A total of 1,650 ng of labeled cRNA was prepared for fragmentation adding 11 μl 10X Blocking Agent and 2.2 μl of 25X Fragmentation Buffer, heated at 60°C for 30 min, and finally diluted by addition with 55 μl 2X GE Hybridization buffer. A volume of 100 μl of hybridization solution was then dispensed in the gasket slide and assembled to the microarray slide (each slide containing four arrays). Slides were incubated for 17 h at 65°C in an Agilent Hybridization Oven, subsequently removed from the hybridization chamber, quickly submerged in GE Wash Buffer 1 to disassembly the slides and then washed in GE Wash Buffer 1 for approximately 1 minute followed by one additional wash in pre-warmed (37°C) GE Wash Buffer 2.

### Data acquisition and analysis

Hybridized slides were scanned at 5 μm resolution using an Agilent G2565BA DNA microarray scanner. Default settings were modified to scan the same slide twice at two different sensitivity levels (XDR Hi 100% and XDR Lo 10%). The two linked images generated were analyzed together and data were extracted and background subtracted using the standard procedures contained in the Agilent Feature Extraction (FE) Software version 9.5.1. The software returns a series of spot quality measures in order to evaluate the goodness and the reliability of spot intensity estimates. All control features (positive, negative, etc.), except for Spike-in (Spike-in Viral RNAs), were excluded from subsequent analyses. Spike-in control intensities were used to identify the best normalization procedure for each dataset. After normalization, spike intensities are expected to be uniform across the experiments of a given dataset. Normalization procedures were performed using R statistical software [71]. Quantile normalization always outperformed cyclic lowess and quantile-normalized data were used in all subsequent analyses.

Statistical tests implemented in the program Significance Analysis of Microarray (SAM) [72] were used to identify differentially expressed genes between digestive gland and gill tissues. The same approach was used to identify differentially expressed genes in both digestive glands and gills between MA and AL sampled individuals.

Pearson correlation coefficients were estimated within and among arrays with Statgraphics Centurion XVI to evaluate repeatability and precision of the obtained microarray data.

### Functional enrichment of differentially expressed genes

Functional annotation analysis of differentially expressed genes was performed using the DAVID (Database for *Annotation*, Visualization and Integrated Discovery) web-server [23].

Functional annotation of differentially expressed genes between gills and digestive glands was achieved using DAVID software. "Biological process", "Molecular function" and "Cellular component" annotation was carried out by setting gene count = 10 and ease = 0.05. KEGG pathway analysis was then performed with gene count = 4 and ease = 0.05. David analyses of differentially expressed genes between Manila clam tissues sampled in Alberoni and Marghera were performed by setting gene count = 2 and ease = 0.1 Since DAVID databases contain functional annotation data for a limited number of species, it was necessary to link *R. philippinarum *transcripts with sequence identifiers that could be recognized in DAVID (Ensembl Human Gene IDs and Ensembl Zebrafish Gene IDs). This was carried out through dedicated Blast searches implemented as follows: i) blastx and blastn options were both used to search significant matches of the Manila clam sequences directly against human Ensembl proteins and transcripts respectively, ii) a first search was performed using either blastn or blastx against all zebrafish Ensembl proteins. Finally, *Homo sapiens *Ensembl Gene IDs were obtained from the corresponding Ensembl protein entries using the BIOMART data mining tool [73].

### Evolutionary analyses

Evolutionary analyses were performed to determine patterns of divergence of the GST genes in *R. philippinarum *and to define putative orthology between GST genes in different species. Protein sequences of GST domains were aligned using TCoffee [74] applying default settings, while GBlock [75] was used to eliminate poorly aligned positions and divergent regions prior to phylogenetic analysis.

GST sequences described from *Homo sapiens, Haliotis discus*, *Unio timidus, Cristaria plicata, Mytilus edulis*, *Mytilus galloprovincialis*, *Danio rerio*, *Dreissena polymorpha*, *Chlamys farreri*, *Corbicula fluminea*, *Mercenaria, mercenaria, Laternula elliptica*, *Mactra veneriformis*, *Cipangopaludina cathayensis *were included in the alignment.

Phylogenetic trees were inferred by the maximum likelihood (ML) method [76] using the Phyml 2.4.4 program [77]. Non-parametric bootstrap resamplings were performed to evaluate the robustness of tree topology.

## Authors' contributions

LB, MM, CC, TM, GC, RL, LC and CS conceived and designed the project. RR produced the EST sequences. SB and AC conceived and constructed the database. MM carried out probe design and editing, and performed microarray experiments. MM and LB executed all statistical analyses. MM performed functional annotation analyses. LB and MM wrote the manuscript. All listed authors edited the manuscript. All authors read and approved the manuscript.

## Supplementary Material

Additional file 1 **Summary of Blastx (E-value < 10-3) and Blastn (E-value < 10-5) similarity searches on several protein and nucleotide databases for R.philippinarum transcripts annotation**. Click here for file

Additional file 2 ***GO terms associated to R. philippinarum transcripts represented in the microarray using "Generic GO slim" in Blast2GO software***. Details about "Biological process", "Molecular function" and "Cellular component" GO terms. Click here for file

Additional file 3 **Lists of annotated differentially expressed genes in digestive gland compared to gills. Score, Fold change, q-value and annotation on several protein and nucleotide database were also reported**. Click here for file

Additional file 4 ***List of significant probes identified by SAM analysis by comparison of digestive gland and gills of Manila clam sampled in Alberoni and Marghera. Significant probes in common between both tissue were also reported***. Down-regulated genes in Marghera samples are highlighted in green while over expressed genes are highlighted in red. For each transcript, fold change, q-value, annotation and *homologs *zebrafish Ensembl Gene IDs (ENDARP and ENDARG) are reported. Click here for file

Additional file 5 ***Sampling site in the Lagoon of Venice***. Map of the Venice Lagoon showing Manila clam sampling sites. Click here for file
