# Supplementary material for: Transcriptome sequencing and microarray development for the Manila clam, Ruditapes philippinarum: genomic tools for environmental monitoring
Source: BMC Genomics. 2011 May 12;12:234. doi: 10.1186/1471-2164-12-234 (PMC3107815; doi:10.1186/1471-2164-12-234)
Supplement: Additional file 2 — GO terms associated to R. philippinarum transcripts represented in the microarray using "Generic GO slim" in Blast2GO software. Details about "Biological process", "Molecular function" and "Cellular component" GO terms. [file 1471-2164-12-234-S2.DOC]

| [**GO:0003674**](http://amigo.geneontology.org/cgi-bin/amigo/go.cgi?action=query&view=details&search_constraint=terms&query=GO:0003674): **Molecular_Function** | | | |
| --- | --- | --- | --- |
| **GO class ID** | **Definition** | **Count** | **Fraction** |
| [GO:0005488](http://amigo.geneontology.org/cgi-bin/amigo/go.cgi?action=query&view=details&search_constraint=terms&query=GO:0005488) | binding | 3400 | 19.51% |
| [GO:0003824](http://amigo.geneontology.org/cgi-bin/amigo/go.cgi?action=query&view=details&search_constraint=terms&query=GO:0003824) | catalytic activity | 1720 | 9.87% |
| [GO:0005515](http://amigo.geneontology.org/cgi-bin/amigo/go.cgi?action=query&view=details&search_constraint=terms&query=GO:0005515) | protein binding | 1551 | 8.90% |
| [GO:0016787](http://amigo.geneontology.org/cgi-bin/amigo/go.cgi?action=query&view=details&search_constraint=terms&query=GO:0016787) | hydrolase activity | 702 | 4.03% |
| [GO:0003676](http://amigo.geneontology.org/cgi-bin/amigo/go.cgi?action=query&view=details&search_constraint=terms&query=GO:0003676) | nucleic acid binding | 561 | 3.22% |
| [GO:0005198](http://amigo.geneontology.org/cgi-bin/amigo/go.cgi?action=query&view=details&search_constraint=terms&query=GO:0005198) | structural molecule activity | 550 | 3.16% |
| [GO:0000166](http://amigo.geneontology.org/cgi-bin/amigo/go.cgi?action=query&view=details&search_constraint=terms&query=GO:0000166) | nucleotide binding | 351 | 2.01% |
| [GO:0016740](http://amigo.geneontology.org/cgi-bin/amigo/go.cgi?action=query&view=details&search_constraint=terms&query=GO:0016740) | transferase activity | 331 | 1.90% |
| [GO:0003723](http://amigo.geneontology.org/cgi-bin/amigo/go.cgi?action=query&view=details&search_constraint=terms&query=GO:0003723) | RNA binding | 295 | 1.69% |
| [GO:0005215](http://amigo.geneontology.org/cgi-bin/amigo/go.cgi?action=query&view=details&search_constraint=terms&query=GO:0005215) | transporter activity | 277 | 1.59% |
| [GO:0030528](http://amigo.geneontology.org/cgi-bin/amigo/go.cgi?action=query&view=details&search_constraint=terms&query=GO:0030528) | transcription regulator activity | 176 | 1.01% |
| [GO:0008233](http://amigo.geneontology.org/cgi-bin/amigo/go.cgi?action=query&view=details&search_constraint=terms&query=GO:0008233) | peptidase activity | 169 | 0.97% |
| [GO:0003677](http://amigo.geneontology.org/cgi-bin/amigo/go.cgi?action=query&view=details&search_constraint=terms&query=GO:0003677) | DNA binding | 164 | 0.94% |
| [GO:0008092](http://amigo.geneontology.org/cgi-bin/amigo/go.cgi?action=query&view=details&search_constraint=terms&query=GO:0008092) | cytoskeletal protein binding | 145 | 0.83% |
| [GO:0016301](http://amigo.geneontology.org/cgi-bin/amigo/go.cgi?action=query&view=details&search_constraint=terms&query=GO:0016301) | kinase activity | 136 | 0.78% |
| [GO:0004871](http://amigo.geneontology.org/cgi-bin/amigo/go.cgi?action=query&view=details&search_constraint=terms&query=GO:0004871) | signal transducer activity | 78 | 0.45% |
| [GO:0004672](http://amigo.geneontology.org/cgi-bin/amigo/go.cgi?action=query&view=details&search_constraint=terms&query=GO:0004672) | protein kinase activity | 77 | 0.44% |
| [GO:0045182](http://amigo.geneontology.org/cgi-bin/amigo/go.cgi?action=query&view=details&search_constraint=terms&query=GO:0045182) | translation regulator activity | 65 | 0.37% |
| [GO:0005509](http://amigo.geneontology.org/cgi-bin/amigo/go.cgi?action=query&view=details&search_constraint=terms&query=GO:0005509) | calcium ion binding | 65 | 0.37% |
| [GO:0005102](http://amigo.geneontology.org/cgi-bin/amigo/go.cgi?action=query&view=details&search_constraint=terms&query=GO:0005102) | receptor binding | 61 | 0.35% |
| [GO:0008135](http://amigo.geneontology.org/cgi-bin/amigo/go.cgi?action=query&view=details&search_constraint=terms&query=GO:0008135) | translation factor activity, nucleic acid binding | 61 | 0.35% |
| [GO:0030234](http://amigo.geneontology.org/cgi-bin/amigo/go.cgi?action=query&view=details&search_constraint=terms&query=GO:0030234) | enzyme regulator activity | 59 | 0.34% |
| [GO:0004872](http://amigo.geneontology.org/cgi-bin/amigo/go.cgi?action=query&view=details&search_constraint=terms&query=GO:0004872) | receptor activity | 58 | 0.33% |
| [GO:0003774](http://amigo.geneontology.org/cgi-bin/amigo/go.cgi?action=query&view=details&search_constraint=terms&query=GO:0003774) | motor activity | 56 | 0.32% |
| [GO:0003779](http://amigo.geneontology.org/cgi-bin/amigo/go.cgi?action=query&view=details&search_constraint=terms&query=GO:0003779) | actin binding | 44 | 0.25% |
| [GO:0003700](http://amigo.geneontology.org/cgi-bin/amigo/go.cgi?action=query&view=details&search_constraint=terms&query=GO:0003700) | transcription factor activity | 43 | 0.25% |
| [GO:0008289](http://amigo.geneontology.org/cgi-bin/amigo/go.cgi?action=query&view=details&search_constraint=terms&query=GO:0008289) | lipid binding | 31 | 0.18% |
| [GO:0030246](http://amigo.geneontology.org/cgi-bin/amigo/go.cgi?action=query&view=details&search_constraint=terms&query=GO:0030246) | carbohydrate binding | 24 | 0.14% |
| [GO:0004721](http://amigo.geneontology.org/cgi-bin/amigo/go.cgi?action=query&view=details&search_constraint=terms&query=GO:0004721) | phosphoprotein phosphatase activity | 20 | 0.11% |
| [GO:0004518](http://amigo.geneontology.org/cgi-bin/amigo/go.cgi?action=query&view=details&search_constraint=terms&query=GO:0004518) | nuclease activity | 20 | 0.11% |
| [GO:0016209](http://amigo.geneontology.org/cgi-bin/amigo/go.cgi?action=query&view=details&search_constraint=terms&query=GO:0016209) | antioxidant activity | 19 | 0.11% |
| [GO:0003682](http://amigo.geneontology.org/cgi-bin/amigo/go.cgi?action=query&view=details&search_constraint=terms&query=GO:0003682) | chromatin binding | 13 | 0.07% |
| [GO:0005216](http://amigo.geneontology.org/cgi-bin/amigo/go.cgi?action=query&view=details&search_constraint=terms&query=GO:0005216) | ion channel activity | 13 | 0.07% |
| [GO:0005326](http://amigo.geneontology.org/cgi-bin/amigo/go.cgi?action=query&view=details&search_constraint=terms&query=GO:0005326) | neurotransmitter transporter activity | 4 | 0.02% |

**MOLECULAR FUNCTION GO terms**

**BIOLOGICAL PROCESS GO terms**

| [**GO:0008150**](http://amigo.geneontology.org/cgi-bin/amigo/go.cgi?action=query&view=details&search_constraint=terms&query=GO:0008150) **Biological_Process** | | | |
| --- | --- | --- | --- |
| **GO class ID** | **Definition** | **Count** | **Fraction** |
| [GO:0008152](http://amigo.geneontology.org/cgi-bin/amigo/go.cgi?action=query&view=details&search_constraint=terms&query=GO:0008152) | metabolism | 2999 | 12.44% |
| [GO:0019538](http://amigo.geneontology.org/cgi-bin/amigo/go.cgi?action=query&view=details&search_constraint=terms&query=GO:0019538) | protein metabolism | 1213 | 5.03% |
| [GO:0009058](http://amigo.geneontology.org/cgi-bin/amigo/go.cgi?action=query&view=details&search_constraint=terms&query=GO:0009058) | biosynthesis | 1118 | 4.64% |
| [GO:0007275](http://amigo.geneontology.org/cgi-bin/amigo/go.cgi?action=query&view=details&search_constraint=terms&query=GO:0007275) | development | 1079 | 4.48% |
| [GO:0016043](http://amigo.geneontology.org/cgi-bin/amigo/go.cgi?action=query&view=details&search_constraint=terms&query=GO:0016043) | cell organization and biogenesis | 915 | 3.80% |
| [GO:0006139](http://amigo.geneontology.org/cgi-bin/amigo/go.cgi?action=query&view=details&search_constraint=terms&query=GO:0006139) | nucleobase, nucleoside, nucleotide and nucleic acid metabolism | 780 | 3.24% |
| [GO:0006412](http://amigo.geneontology.org/cgi-bin/amigo/go.cgi?action=query&view=details&search_constraint=terms&query=GO:0006412) | protein biosynthesis | 659 | 2.73% |
| [GO:0006810](http://amigo.geneontology.org/cgi-bin/amigo/go.cgi?action=query&view=details&search_constraint=terms&query=GO:0006810) | transport | 634 | 2.63% |
| [GO:0006996](http://amigo.geneontology.org/cgi-bin/amigo/go.cgi?action=query&view=details&search_constraint=terms&query=GO:0006996) | organelle organization and biogenesis | 465 | 1.93% |
| [GO:0009653](http://amigo.geneontology.org/cgi-bin/amigo/go.cgi?action=query&view=details&search_constraint=terms&query=GO:0009653) | morphogenesis | 426 | 1.77% |
| [GO:0007154](http://amigo.geneontology.org/cgi-bin/amigo/go.cgi?action=query&view=details&search_constraint=terms&query=GO:0007154) | cell communication | 390 | 1.62% |
| [GO:0030154](http://amigo.geneontology.org/cgi-bin/amigo/go.cgi?action=query&view=details&search_constraint=terms&query=GO:0030154) | cell differentiation | 372 | 1.54% |
| [GO:0007049](http://amigo.geneontology.org/cgi-bin/amigo/go.cgi?action=query&view=details&search_constraint=terms&query=GO:0007049) | cell cycle | 366 | 1.52% |
| [GO:0006950](http://amigo.geneontology.org/cgi-bin/amigo/go.cgi?action=query&view=details&search_constraint=terms&query=GO:0006950) | response to stress | 315 | 1.31% |
| [GO:0007165](http://amigo.geneontology.org/cgi-bin/amigo/go.cgi?action=query&view=details&search_constraint=terms&query=GO:0007165) | signal transduction | 293 | 1.22% |
| [GO:0009056](http://amigo.geneontology.org/cgi-bin/amigo/go.cgi?action=query&view=details&search_constraint=terms&query=GO:0009056) | catabolism | 259 | 1.07% |
| [GO:0000003](http://amigo.geneontology.org/cgi-bin/amigo/go.cgi?action=query&view=details&search_constraint=terms&query=GO:0000003) | reproduction | 243 | 1.01% |
| [GO:0009790](http://amigo.geneontology.org/cgi-bin/amigo/go.cgi?action=query&view=details&search_constraint=terms&query=GO:0009790) | embryonic development | 243 | 1.01% |
| [GO:0007010](http://amigo.geneontology.org/cgi-bin/amigo/go.cgi?action=query&view=details&search_constraint=terms&query=GO:0007010) | cytoskeleton organization and biogenesis | 242 | 1.00% |
| [GO:0006350](http://amigo.geneontology.org/cgi-bin/amigo/go.cgi?action=query&view=details&search_constraint=terms&query=GO:0006350) | transcription | 239 | 0.99% |
| [GO:0016265](http://amigo.geneontology.org/cgi-bin/amigo/go.cgi?action=query&view=details&search_constraint=terms&query=GO:0016265) | death | 221 | 0.92% |
| [GO:0008219](http://amigo.geneontology.org/cgi-bin/amigo/go.cgi?action=query&view=details&search_constraint=terms&query=GO:0008219) | cell death | 221 | 0.92% |
| [GO:0006091](http://amigo.geneontology.org/cgi-bin/amigo/go.cgi?action=query&view=details&search_constraint=terms&query=GO:0006091) | generation of precursor metabolites and energy | 215 | 0.89% |
| [GO:0006464](http://amigo.geneontology.org/cgi-bin/amigo/go.cgi?action=query&view=details&search_constraint=terms&query=GO:0006464) | protein modification | 208 | 0.86% |
| [GO:0040007](http://amigo.geneontology.org/cgi-bin/amigo/go.cgi?action=query&view=details&search_constraint=terms&query=GO:0040007) | growth | 179 | 0.74% |
| [GO:0005975](http://amigo.geneontology.org/cgi-bin/amigo/go.cgi?action=query&view=details&search_constraint=terms&query=GO:0005975) | carbohydrate metabolism | 150 | 0.62% |
| [GO:0006811](http://amigo.geneontology.org/cgi-bin/amigo/go.cgi?action=query&view=details&search_constraint=terms&query=GO:0006811) | ion transport | 146 | 0.61% |
| [GO:0006629](http://amigo.geneontology.org/cgi-bin/amigo/go.cgi?action=query&view=details&search_constraint=terms&query=GO:0006629) | lipid metabolism | 135 | 0.56% |
| [GO:0009605](http://amigo.geneontology.org/cgi-bin/amigo/go.cgi?action=query&view=details&search_constraint=terms&query=GO:0009605) | response to external stimulus | 129 | 0.54% |
| [GO:0015031](http://amigo.geneontology.org/cgi-bin/amigo/go.cgi?action=query&view=details&search_constraint=terms&query=GO:0015031) | protein transport | 126 | 0.52% |
| [GO:0008283](http://amigo.geneontology.org/cgi-bin/amigo/go.cgi?action=query&view=details&search_constraint=terms&query=GO:0008283) | cell proliferation | 103 | 0.43% |
| [GO:0009607](http://amigo.geneontology.org/cgi-bin/amigo/go.cgi?action=query&view=details&search_constraint=terms&query=GO:0009607) | response to biotic stimulus | 101 | 0.42% |
| [GO:0019725](http://amigo.geneontology.org/cgi-bin/amigo/go.cgi?action=query&view=details&search_constraint=terms&query=GO:0019725) | cell homeostasis | 100 | 0.41% |
| [GO:0007610](http://amigo.geneontology.org/cgi-bin/amigo/go.cgi?action=query&view=details&search_constraint=terms&query=GO:0007610) | behavior | 94 | 0.39% |
| [GO:0006259](http://amigo.geneontology.org/cgi-bin/amigo/go.cgi?action=query&view=details&search_constraint=terms&query=GO:0006259) | DNA metabolism | 93 | 0.39% |
| [GO:0007267](http://amigo.geneontology.org/cgi-bin/amigo/go.cgi?action=query&view=details&search_constraint=terms&query=GO:0007267) | cell-cell signaling | 82 | 0.34% |
| [GO:0009628](http://amigo.geneontology.org/cgi-bin/amigo/go.cgi?action=query&view=details&search_constraint=terms&query=GO:0009628) | response to abiotic stimulus | 70 | 0.29% |
| [GO:0006519](http://amigo.geneontology.org/cgi-bin/amigo/go.cgi?action=query&view=details&search_constraint=terms&query=GO:0006519) | amino acid and derivative metabolism | 52 | 0.22% |
| [GO:0009719](http://amigo.geneontology.org/cgi-bin/amigo/go.cgi?action=query&view=details&search_constraint=terms&query=GO:0009719) | response to endogenous stimulus | 48 | 0.20% |
| [GO:0016049](http://amigo.geneontology.org/cgi-bin/amigo/go.cgi?action=query&view=details&search_constraint=terms&query=GO:0016049) | cell growth | 36 | 0.15% |
| [GO:0007005](http://amigo.geneontology.org/cgi-bin/amigo/go.cgi?action=query&view=details&search_constraint=terms&query=GO:0007005) | mitochondrion organization and biogenesis | 28 | 0.12% |
| [GO:0008037](http://amigo.geneontology.org/cgi-bin/amigo/go.cgi?action=query&view=details&search_constraint=terms&query=GO:0008037) | cell recognition | 23 | 0.10% |
| [GO:0040029](http://amigo.geneontology.org/cgi-bin/amigo/go.cgi?action=query&view=details&search_constraint=terms&query=GO:0040029) | regulation of gene expression, epigenetic | 21 | 0.09% |
| [GO:0016032](http://amigo.geneontology.org/cgi-bin/amigo/go.cgi?action=query&view=details&search_constraint=terms&query=GO:0016032) | viral life cycle | 19 | 0.08% |
| [GO:0019748](http://amigo.geneontology.org/cgi-bin/amigo/go.cgi?action=query&view=details&search_constraint=terms&query=GO:0019748) | secondary metabolism | 7 | 0.03% |
| [GO:0007028](http://amigo.geneontology.org/cgi-bin/amigo/go.cgi?action=query&view=details&search_constraint=terms&query=GO:0007028) | cytoplasm organization and biogenesis | 1 | 0.00% |

**CELLULAR COMPONENT GO terms**

| [**GO:0005575**](http://amigo.geneontology.org/cgi-bin/amigo/go.cgi?action=query&view=details&search_constraint=terms&query=GO:0005575) **Cellular_Component** | | | |
| --- | --- | --- | --- |
| **GO class ID** | **Definition** | **Count** | **Fraction** |
| [GO:0005623](http://amigo.geneontology.org/cgi-bin/amigo/go.cgi?action=query&view=details&search_constraint=terms&query=GO:0005623) | cell | 5114 | 24.34% |
| [GO:0005622](http://amigo.geneontology.org/cgi-bin/amigo/go.cgi?action=query&view=details&search_constraint=terms&query=GO:0005622) | intracellular | 3956 | 18.83% |
| [GO:0005737](http://amigo.geneontology.org/cgi-bin/amigo/go.cgi?action=query&view=details&search_constraint=terms&query=GO:0005737) | cytoplasm | 2404 | 11.44% |
| [GO:0005634](http://amigo.geneontology.org/cgi-bin/amigo/go.cgi?action=query&view=details&search_constraint=terms&query=GO:0005634) | nucleus | 714 | 3.40% |
| [GO:0005829](http://amigo.geneontology.org/cgi-bin/amigo/go.cgi?action=query&view=details&search_constraint=terms&query=GO:0005829) | cytosol | 632 | 3.01% |
| [GO:0005840](http://amigo.geneontology.org/cgi-bin/amigo/go.cgi?action=query&view=details&search_constraint=terms&query=GO:0005840) | ribosome | 557 | 2.65% |
| [GO:0005739](http://amigo.geneontology.org/cgi-bin/amigo/go.cgi?action=query&view=details&search_constraint=terms&query=GO:0005739) | mitochondrion | 474 | 2.26% |
| [GO:0005856](http://amigo.geneontology.org/cgi-bin/amigo/go.cgi?action=query&view=details&search_constraint=terms&query=GO:0005856) | cytoskeleton | 420 | 2.00% |
| [GO:0005886](http://amigo.geneontology.org/cgi-bin/amigo/go.cgi?action=query&view=details&search_constraint=terms&query=GO:0005886) | plasma membrane | 265 | 1.26% |
| [GO:0005576](http://amigo.geneontology.org/cgi-bin/amigo/go.cgi?action=query&view=details&search_constraint=terms&query=GO:0005576) | extracellular region | 171 | 0.81% |
| [GO:0005654](http://amigo.geneontology.org/cgi-bin/amigo/go.cgi?action=query&view=details&search_constraint=terms&query=GO:0005654) | nucleoplasm | 147 | 0.70% |
| [GO:0005811](http://amigo.geneontology.org/cgi-bin/amigo/go.cgi?action=query&view=details&search_constraint=terms&query=GO:0005811) | lipid particle | 118 | 0.56% |
| [GO:0005783](http://amigo.geneontology.org/cgi-bin/amigo/go.cgi?action=query&view=details&search_constraint=terms&query=GO:0005783) | endoplasmic reticulum | 100 | 0.48% |
| [GO:0005730](http://amigo.geneontology.org/cgi-bin/amigo/go.cgi?action=query&view=details&search_constraint=terms&query=GO:0005730) | nucleolus | 91 | 0.43% |
| [GO:0005694](http://amigo.geneontology.org/cgi-bin/amigo/go.cgi?action=query&view=details&search_constraint=terms&query=GO:0005694) | chromosome | 77 | 0.37% |
| [GO:0005815](http://amigo.geneontology.org/cgi-bin/amigo/go.cgi?action=query&view=details&search_constraint=terms&query=GO:0005815) | microtubule organizing center | 48 | 0.23% |
| [GO:0005794](http://amigo.geneontology.org/cgi-bin/amigo/go.cgi?action=query&view=details&search_constraint=terms&query=GO:0005794) | Golgi apparatus | 47 | 0.22% |
| [GO:0016023](http://amigo.geneontology.org/cgi-bin/amigo/go.cgi?action=query&view=details&search_constraint=terms&query=GO:0016023) | cytoplasmic membrane-bound vesicle | 43 | 0.20% |
| [GO:0005578](http://amigo.geneontology.org/cgi-bin/amigo/go.cgi?action=query&view=details&search_constraint=terms&query=GO:0005578) | extracellular matrix (sensu Metazoa) | 35 | 0.17% |
| [GO:0005773](http://amigo.geneontology.org/cgi-bin/amigo/go.cgi?action=query&view=details&search_constraint=terms&query=GO:0005773) | vacuole | 34 | 0.16% |
| [GO:0005615](http://amigo.geneontology.org/cgi-bin/amigo/go.cgi?action=query&view=details&search_constraint=terms&query=GO:0005615) | extracellular space | 33 | 0.16% |
| [GO:0005764](http://amigo.geneontology.org/cgi-bin/amigo/go.cgi?action=query&view=details&search_constraint=terms&query=GO:0005764) | lysosome | 27 | 0.13% |
| [GO:0005768](http://amigo.geneontology.org/cgi-bin/amigo/go.cgi?action=query&view=details&search_constraint=terms&query=GO:0005768) | endosome | 20 | 0.10% |
| [GO:0000228](http://amigo.geneontology.org/cgi-bin/amigo/go.cgi?action=query&view=details&search_constraint=terms&query=GO:0000228) | nuclear chromosome | 20 | 0.10% |
| [GO:0005635](http://amigo.geneontology.org/cgi-bin/amigo/go.cgi?action=query&view=details&search_constraint=terms&query=GO:0005635) | nuclear membrane | 14 | 0.07% |
| [GO:0005929](http://amigo.geneontology.org/cgi-bin/amigo/go.cgi?action=query&view=details&search_constraint=terms&query=GO:0005929) | cilium | 10 | 0.05% |
| [GO:0005777](http://amigo.geneontology.org/cgi-bin/amigo/go.cgi?action=query&view=details&search_constraint=terms&query=GO:0005777) | peroxisome | 9 | 0.04% |
| [GO:0009536](http://amigo.geneontology.org/cgi-bin/amigo/go.cgi?action=query&view=details&search_constraint=terms&query=GO:0009536) | plastid | 7 | 0.03% |
| [GO:0030312](http://amigo.geneontology.org/cgi-bin/amigo/go.cgi?action=query&view=details&search_constraint=terms&query=GO:0030312) | external encapsulating structure | 5 | 0.02% |
| [GO:0005618](http://amigo.geneontology.org/cgi-bin/amigo/go.cgi?action=query&view=details&search_constraint=terms&query=GO:0005618) | cell wall | 5 | 0.02% |
