# Supplementary figures and images for: Transcriptome sequencing and microarray development for the Manila clam, Ruditapes philippinarum: genomic tools for environmental monitoring
Source: BMC Genomics. 2011 May 12;12:234. doi: 10.1186/1471-2164-12-234 (PMC3107815; doi:10.1186/1471-2164-12-234)

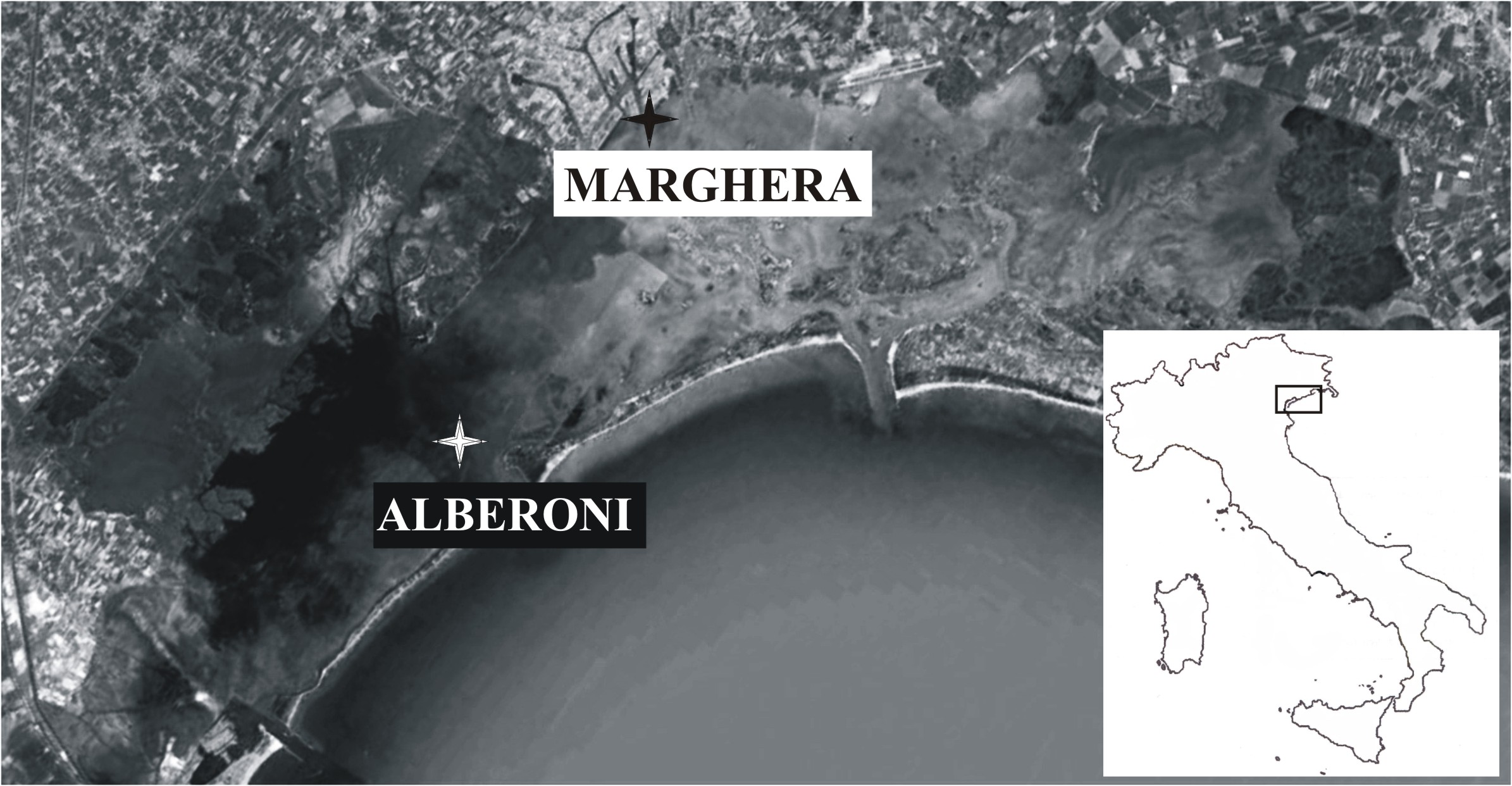

Supplement: Additional file 5 — Sampling site in the Lagoon of Venice. Map of the Venice Lagoon showing Manila clam sampling sites. [file 1471-2164-12-234-S5.JPEG]
